# Unraveling the function and structure impact of deleterious missense SNPs in the human OX1R receptor by computational analysis

**DOI:** 10.1038/s41598-023-49809-4

**Published:** 2024-01-08

**Authors:** Mahvash Farajzadeh-Dehkordi, Ladan Mafakher, Abbas Harifi, Hashem Haghdoost-Yazdi, Hossein Piri, Babak Rahmani

**Affiliations:** 1 Student Research Committee, Qazvin University of Medical Sciences, Qazvin, Iran; 2 Department of Molecular Medicine, Qazvin University of Medical Sciences, Qazvin, Iran; 3 Thalassemia & Hemoglobinopathy Research Center, Health Research Institute, Ahvaz Jundishapur University of Medical Sciences, Ahvaz, Iran; 4 Department of Electrical and Computer Engineering, University of Hormozgan, Bandar Abbas, Hormozgan Iran; 5 Cellular and Molecular Research Center, Research Institute for Prevention of Non-Communicable Disease, Qazvin University of Medical Sciences, Qazvin, Iran

**Keywords:** Biochemistry, Computational biology and bioinformatics, Genetics, Diseases, Molecular medicine

## Abstract

The orexin/hypocretin receptor type 1 (OX1R) plays a crucial role in regulating various physiological functions, especially feeding behavior, addiction, and reward. Genetic variations in the OX1R have been associated with several neurological disorders. In this study, we utilized a combination of sequence and structure-based computational tools to identify the most deleterious missense single nucleotide polymorphisms (SNPs) in the *OX1R* gene. Our findings revealed four highly conserved and structurally destabilizing missense SNPs, namely R144C, I148N, S172W, and A297D, located in the GTP-binding domain. Molecular dynamics simulations analysis demonstrated that all four most detrimental mutant proteins altered the overall structural flexibility and dynamics of OX1R protein, resulting in significant changes in the structural organization and motion of the protein. These findings provide valuable insights into the impact of missense SNPs on OX1R function loss and their potential contribution to the development of neurological disorders, thereby guiding future research in this field.

## Introduction

The G protein-coupled orexin/hypocretin receptor type 1 (OX1R/HCRTR1), an integral component of the orexinergic system, is widely acknowledged for its important role in promoting healthy life^1,2^.

The human *OX1R* gene is located on chromosome 1p35.2 and encodes a 425 amino acid protein^3^. The protein comprises seven helical transmembrane segments (TM1–7) linked by three extracellular and intracellular loops (ECL1–3 and ICL1–3), an intracellular C-terminus, and an extracellular N-terminus^4^.

Numerous studies have demonstrated that OX1R exhibits a relatively selective binding affinity for the hypothalamic neuropeptide orexin-A and a lower affinity for the orexin-B neuropeptide^3^. This binding stimulates diverse downstream signaling pathways and contributes to multiple physiological processes, including feeding behavior, breathing, sleep–wake rhythm, drug addiction, reward-seeking, arousal and motivation, nociception, energy homeostasis, stress, and the fight/flight response^3,5–14^. Furthermore, the orexin system has been suggested to possess innate proapoptotic activity, and systemic orexin receptors exhibit neuroprotective and anti-inflammatory effects^15–17^. In addition, neuropeptides and orexin receptors are involved in coordinating sympathetic and cardiovascular activities^18,19^. In contrast, dysregulated OX1R signaling influences pathological conditions such as anxiolytic behaviors, narcolepsy, cataplexy, ischemic stroke, depression, attention deficit and hyperactivity disorder, panic-related anxiety, Alzheimer’s dementia, cancer, and Parkinson’s disease^20–30^. Moreover, genetic polymorphisms in the human OX1R gene are associated with panic disorder, polydipsia-hyponatremia, major depressive disorder (MDD), chronic migraine, aggressiveness, and sleep disorders^31–35^.

Single nucleotide polymorphisms (SNPs) represent a prevalent and stable type of genetic variation within the human genome. They not only serve as valuable biological markers but can also be linked to the development of complex diseases, abnormalities, and variations in drug responses^36–40^. Nonsynonymous SNPs (nsSNPs) in protein-coding regions, which result in alterations to amino acid sequences and the potential creation of mutated proteins with new structural and functional properties, have garnered significant interest. Deleterious nsSNPs at the genomic and/or proteome levels can induce detrimental functions by destabilizing protein tertiary structures, altering the physicochemical characteristics of proteins, and modifying protein–protein interactions, ultimately posing threats to cellular structural integrity^41,42^.

Given the role of OX1R in neuropathological processes, it is crucial to analyze the implications of its nsSNPs. One significant aim in identifying deleterious nsSNPs is to conduct functional and structural evaluations. A comprehensive understanding of the conformational changes experienced by a protein can provide valuable insights into the underlying mechanisms of disease phenotypes and facilitate the identification of potential therapeutic agents capable of modulating protein function^42,43^.

Experimental investigation of the impact of various nsSNPs is an expensive, time-consuming, and challenging process. However, employing in silico analysis through web-based computational methods is a feasible, effective, and affordable approach for investigating numerous SNPs in specific genes^44,45^.

Therefore, the objective of this study is to retrieve OX1R nsSNPs and narrow them to ascertain the deleterious SNPs and assess their pathogenic effects on the protein using various biophysics-based computational algorithms, along with molecular simulation, to gain a better understanding of the mutation effects under physiological conditions.

Overall, the findings of this study will aid in determining the genotype–phenotype association of diseases, contribute to identifying the most significant SNPs in OX1R for population-based research, and be valuable in designing personalized medicine-based treatments for disorders triggered by these genomic SNPs.

## Result

### Distribution of OX1R SNPs

**Figure 1:**
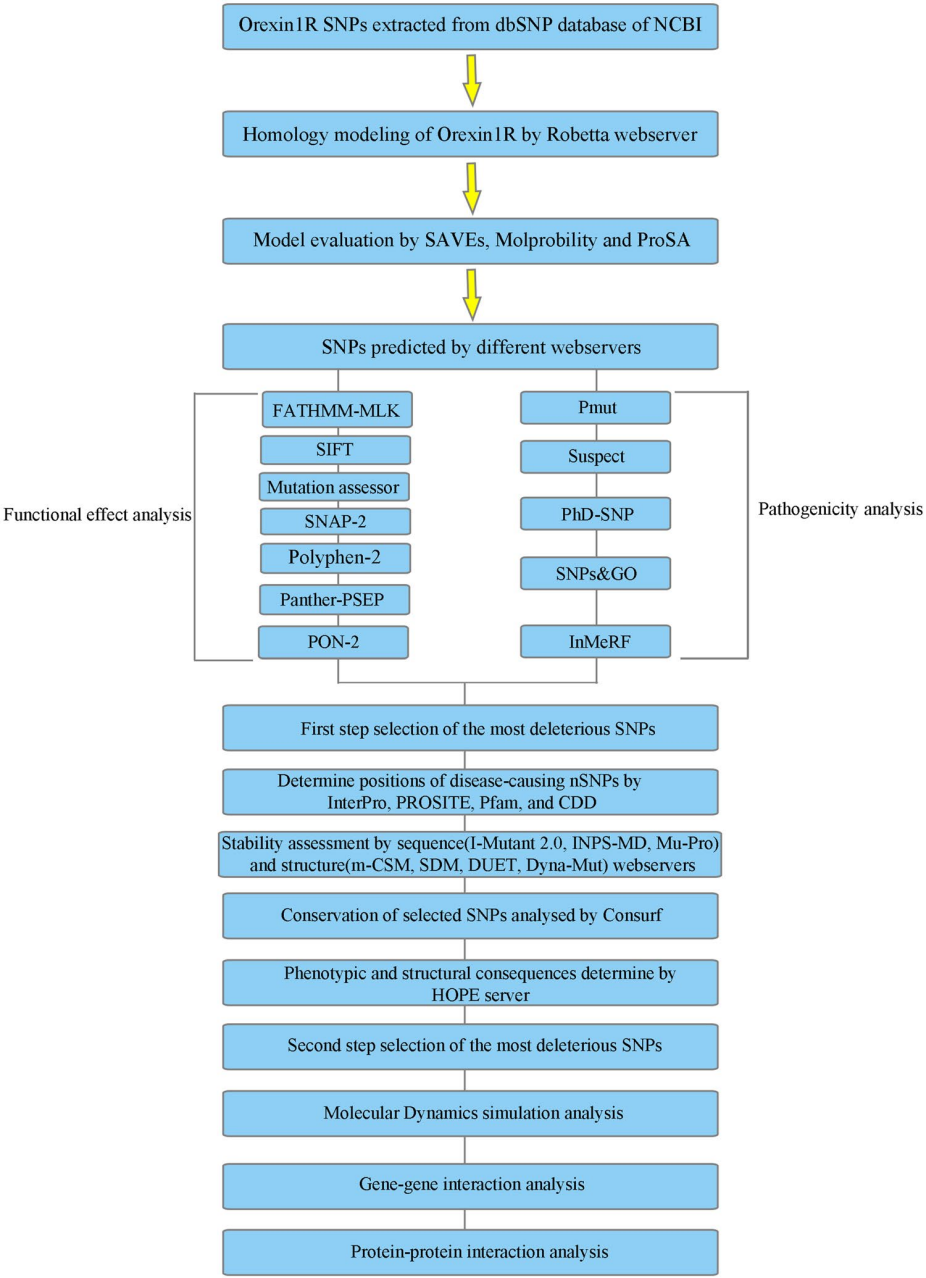
The methodological approach is shown in the schematic diagram.

In the *OX1R* gene, 4295 SNPs were identified (accessed October 22, 2022). These included 2908 intron variants, 177 synonymous variants, 329 missense SNPs, 17 nonsense (stop-gained) SNPs, 219 variants were located in 3′-untranslated regions (UTR), and 237 variants were located in 5′-UTR, in addition to other upstream and downstream variants. Only missense SNPs were selected for analysis and screening. The methodological approach is shown in the schematic diagram (Fig. 1).

### Determining 3D protein structure

**Figure 2:**
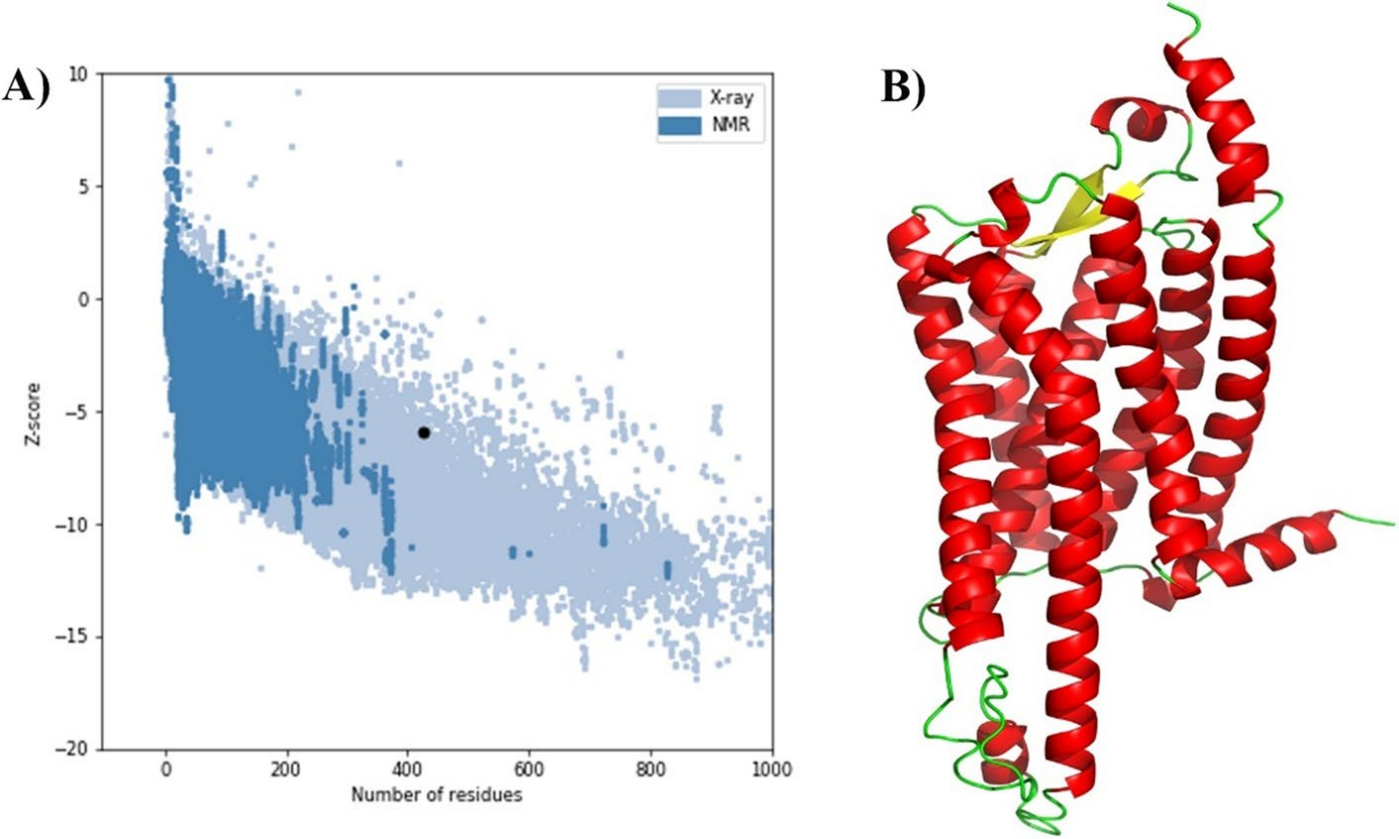
The ProSA plot and tertiary structure of human OX1R protein. (**A**) The ProSA plot of human OX1R protein (black dot) indicates comparable quality of X-ray and NMR. (**B**) The tertiary structure of human OX1R protein predicted by the PyMOL (Red: alpha helix; Yellow: beta-sheet; Green: random coils and other structures).

As the complete structure of the human OX1R protein did not exist in the PDB bank (Fig. S1), homology modeling of the OX1R was performed by the Robetta webserver. Several approaches were used to evaluate the quality of the model (Table S1). Based on the Ramachandran plot analysis using the Procheck web server, more than 90% of the amino acid residues were located in the core and generally allowed regions, indicating that the protein structure is of high quality. The ERRAT value of more than 95% showed the high resolution of the protein model. The verify3D score of 42% demonstrated moderate structural quality for the protein. A Molprobility score of 100% confirms the high resolution of protein structure compared to proposed structural characterizations based on X-rays. The ProSA plot showed the model has a z-score value (− 5.9) in the range of proteins with the same length whose structures were identified by X-ray and NMR structure (Fig. 2A). Comparing the quality of OX1R homology modeling with its crystal structure (PDB ID: 6 to 7) revealed that this model had approximately the same quality as its crystal structure. The tertiary structure of the human OX1R model is shown in Fig. 2B. The structure of OX1R homology modeling and 6 to 7 represented high compatibility (Fig. S2).

### Determining the pathogenic SNPs

To estimate the deleterious SNPs of OX1R, various computational tools were employed. Initially, 329 missense SNPs were assessed using 10 bioinformatics tools: FATHMM-MLK, SIFT, Mutation Assessor, SNAP-2, PolyPhen-2, Panther-PSEP, PON-P2, CADD, Align-GVGD, and VEST-4, to investigate their functional impacts. Among them, 21 missense SNPs were consistently predicted to have functional effects by all the utilized methods (Table S2). Subsequently, the selected SNPs were passed through five different servers, namely Pmut, Suspect, PhD-SNP, SNPs&GO, and InMeRF to evaluate their potential association with pathogenicity. Among the 21 evaluated missense SNPs, 17 SNPs were identified as disease-associated or pathogenic by at least four of the five pathogenicity prediction tools (Table 1). These 17 missense SNPs were selected for further analysis and screening.

**Table 1:** Screening of 21 deleterious missense SNPs associated with OX1R protein.

SNP ID	Protein change	Pmut	Suspect	PhD-SNP	SNPs&GO	InMeRF
rs768118872	I85T	Pathogenic	Pathogenic	Neutral	Disease	Normal
rs1368005577	**N87Y**	**Pathogenic**	**Neutral**	**Disease**	**Disease**	**Pathogenic**
rs1307483513	**S89P**	**Pathogenic**	**Pathogenic**	**Disease**	**Disease**	**Pathogenic**
rs188180909	L90Q	Pathogenic	Pathogenic	Neutral	Disease	Normal
rs778134490	**D92G**	**Pathogenic**	**Pathogenic**	**Disease**	**Disease**	**Pathogenic**
	D92V	Pathogenic	Neutral	Neutral	Disease	Pathogenic
rs1487077579	**A141V**	**Pathogenic**	**Pathogenic**	**Neutral**	**Disease**	**Pathogenic**
rs778794988	**R144C**	**Pathogenic**	**Pathogenic**	**Neutral**	**Disease**	**Pathogenic**
	R144L	Pathogenic	Neutral	Neutral	Disease	Pathogenic
rs201925923	**A147D**	**Pathogenic**	**Pathogenic**	**Disease**	**Disease**	**Pathogenic**
rs1160159351	**I148N**	**Pathogenic**	**Neutral**	**Disease**	**Disease**	**Pathogenic**
rs200634924	**P151L**	**Pathogenic**	**Pathogenic**	**Disease**	**Disease**	**Pathogenic**
rs1239300181	**A161D**	**Pathogenic**	**Pathogenic**	**Disease**	**Disease**	**Pathogenic**
rs746566291	**S172W**	**Pathogenic**	**Pathogenic**	**Disease**	**Disease**	**Pathogenic**
rs201360185	**R293W**	**Pathogenic**	**Pathogenic**	**Disease**	**Disease**	**Pathogenic**
rs767939002	**A297D**	**Pathogenic**	**Pathogenic**	**Neutral**	**Disease**	**Pathogenic**
rs1291095889	**L312Q**	**Pathogenic**	**Pathogenic**	**Neutral**	**Disease**	**Pathogenic**
rs746884559	**P313H**	**Pathogenic**	**Neutral**	**Disease**	**Disease**	**Pathogenic**
	**P313L**	**Pathogenic**	**Neutral**	**Disease**	**Disease**	**Pathogenic**
rs958763496	**S351R**	**Pathogenic**	**Neutral**	**Disease**	**Disease**	**Pathogenic**
rs1479886483	**N354K**	**Pathogenic**	**Neutral**	**Disease**	**Disease**	**Pathogenic**

Boldly highlighted mutations are found to be disease-associated or pathogenic by at least four out of five pathogenicity prediction tools.

### Determining the domain of OX1R protein

We used four tools, InterPro, PROSITE, Pfam, and CDD, to determine the conserved domains and locations of 17 pathogenic missense SNPs in the OX1R protein. All three databases illustrated the existence of an important domain: a seven-helix transmembrane G-protein-coupled receptor (GPCR) domain at positions 63–358, whereas CDD predicted the same domain at positions 47–369. Furthermore, the positions of the 17 pathogenic missense SNPs were analyzed, and all variants were found to be located in the GTP-binding protein domain.

### Determining SNPs’ impact on the OX1R protein stability

The free energy change (ΔΔG) of the native and mutated forms of a protein is a vital index for protein stability. By evaluating the effect of mutations on the free energy, the impact of single-site mutations on protein stability can be precisely identified. A ΔΔG score < 0 indicated a decrease in protein stability, whereas a score > 0 indicated the opposite result.

Of the 17 pathogenic missense SNPs, 12 and 15 SNPs were determined to decrease OX1R protein stability using I-Mutant 2.0, and INPS-MD tools, respectively. In contrast, the Mu-Pro server showed a decreasing effect on all the pathogenic SNPs (Table 2). The m-CSM, SDM, and DUET servers predicted 15, 13, and 14 SNPs, respectively, to be deleterious, as they caused a decrease in the stability of the OX1R protein (Table 2). According to the Dyna-Mut tool, seven SNPs of the 17 pathogenic SNPs had negative ΔΔG values, indicating that they reduced the stability of the OX1R protein (Table 2). Finally, five missense SNPs (R144C, I148N, S172W, A297D, and L312Q) among 17 pathogenic SNPs were identified as the most deleterious to OX1R protein stability (ΔΔG values less than zero) using all seven above-mentioned tools. We selected these five destabilizing SNPs for the phylogenetic conservation analysis.

**Table 2:** Stability prediction analysis of 17 pathogenic missense SNPs of OX1R protein.

SNP ID	Protein change	I-Mutant 2.0	INPS-MD	Mu-Pro	m-CSM	SDM	DUET	Dyna-Mut
rs1368005577	N87Y	I	I	D	D	I	D	I
rs1307483513	S89P	D	D	D	I	D	D	I
rs778134490	D92G	D	D	D	D	D	I	I
rs1487077579	A141V	I	D	D	D	D	D	I
rs778794988	**R144C**	**D**	**D**	**D**	**D**	**D**	**D**	**D**
rs201925923	A147D	I	D	D	D	D	D	I
rs1160159351	**I148N**	**D**	**D**	**D**	**D**	**D**	**D**	**D**
rs200634924	P151L	D	D	D	D	I	D	I
rs1239300181	A161D	I	D	D	D	D	D	D
rs746566291	**S172W**	**D**	**D**	**D**	**D**	**D**	**D**	**D**
rs201360185	R293W	D	D	D	D	D	D	I
rs767939002	**A297D**	**D**	**D**	**D**	**D**	**D**	**D**	**D**
rs1291095889	**L312Q**	**D**	**D**	**D**	**D**	**D**	**D**	**D**
rs746884559	P313H	D	D	D	D	I	D	I
	P313L	D	D	D	D	I	I	I
rs958763496	S351R	I	D	D	D	D	D	I
rs1479886483	N354K	D	I	D	D	D	I	D

Boldly highlighted mutations are found to be destabilizing from all seven tools. Abbreviations: I (Increase) and D (Deceased) of stability.

### Determining the phylogenetically conserved residues in the OX1R protein

The ConSurf tool was used to predict the level of evolutionary conservation at all the residue positions in OX1R. The OX1R protein structure was submitted as an input and the results are shown in Fig. S3. Based on the ConSurf outcomes for the OX1R protein, this protein is more evolutionarily conserved, with a greater number of conserved residues; 158 amino acid positions were estimated to have a conservation score between 7 and 9, and 93 of them had a conservation score of 9 (highly conserved residues). Furthermore, ConSurf analysis determined that out of the five destabilizing missense SNPs SNPs (R144C, I148N, S172W, A297D, and L312Q), three (R144C, I148N, and A297D) were highly conserved (score 9). Owing to their high conservation, residues I148 and A297 were classified as structural and buried sites, while residue R144 was predicted to be a functional and exposed site of the OX1R protein. In addition, ConSurf estimated that S172W had a conservation score of 8 (conserved) and L312Q was found in an intermediately conserved site (score 6). Taken together, we selected four missense SNPs namely, R144C, I148N, S172W, and A297D that were located in conserved positions (score between 7 and 9), and the SNP in the L312 position was excluded from subsequent evaluations because it was revealed to have an intermediately conserved profile (score 6).

### Determining the molecular and phenotypic effects of the nsSNPs

We used the HOPE server to calculate the physicochemical properties of the four most detrimental missense SNPs (R144C, I148N, S172W, and A297D) in OX1R.

In the case of the R144C mutation, the substitution of a large positively charged residue, arginine, with a smaller neutrally charged residue, cysteine, at position 144 occurs. The wild-type residue is found in a domain that is essential for protein activity, but the mutation presents an amino acid with different characteristics, which can interrupt this domain and restrict its function. Furthermore, the analysis reveals that the wild-type residue, arginine, engages in hydrogen bonding and salt bridge interactions with aspartic acid at position 143. Conversely, the substituted residue, cysteine, is unable to establish the same interactions as arginine, resulting in a perturbation of the local stability of the protein.

**Figure 3:**
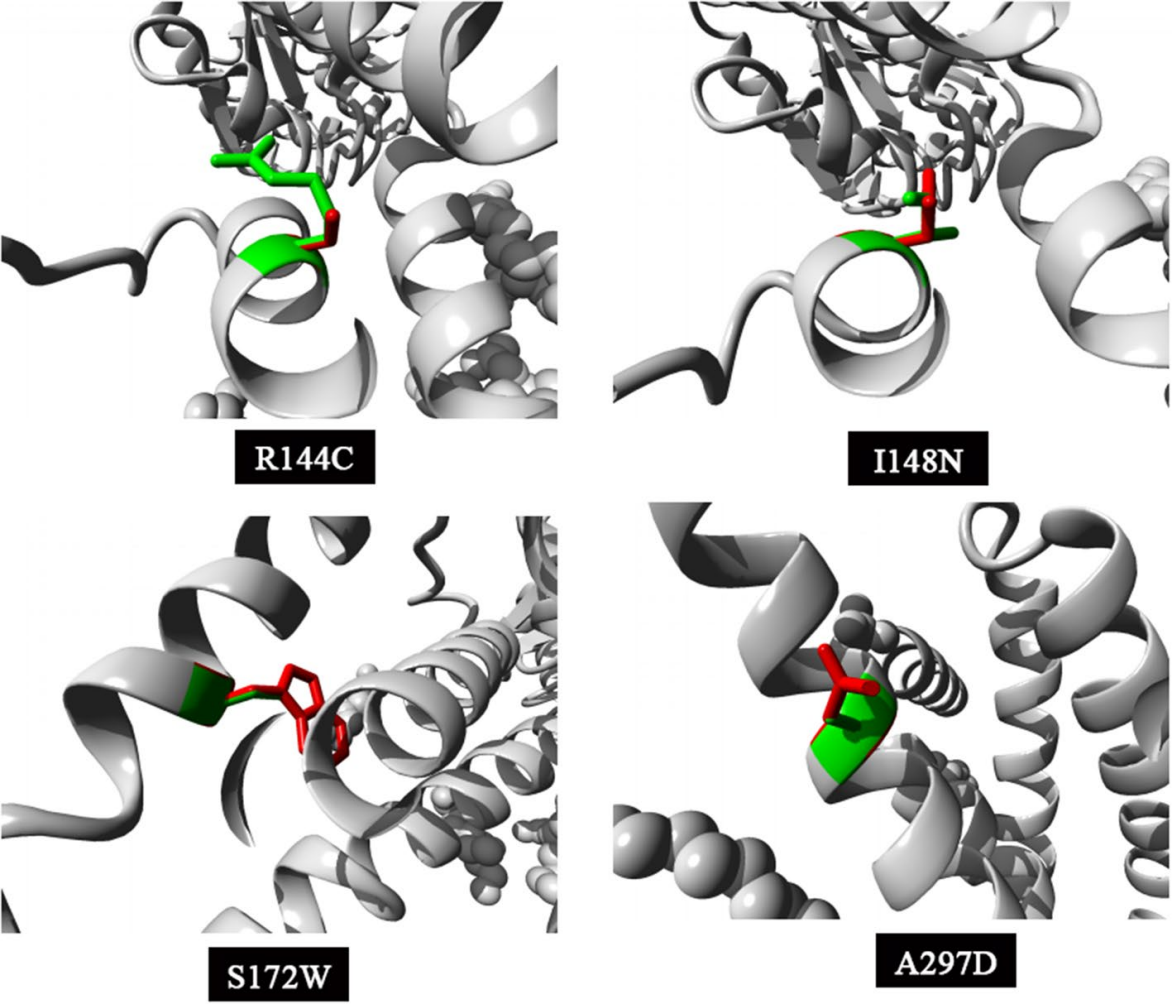
Visualization of wild-type (green) and mutated (red) amino acid residues for the four most detrimental missense SNPs (R144C, I148N, S172W, and A297D) using HOPE Server.

The I148N, S172W, and A297D substitutions presented a larger residue than the wild-type moiety, which likely did not fit into the protein core. If the replaced amino acid does not fit the protein, it causes structural changes that are sometimes hazardous. Particularly, with S172W, it was predicted that the wild-type residue is found in a region annotated in the UniProt database as a transmembrane domain and forms hydrogen bonds with serine at position 131 and isoleucine at position 168, which would affect contact with the lipid membrane and cause loss of the same hydrogen bonds. Moreover, the wild-type residues I148N, S172W, and A297D were more hydrophobic than the mutant residues, which could have caused repulsive hydrophobic interactions in the core of the protein. The A297D substitution introduces a negatively charged residue (aspartic acid) in a buried moiety (alanine), which can lead to protein-folding problems. The A297D in the 3D structure was located in an α-helix. The difference in properties between aspartic acid and alanine can easily cause alanine to not prefer α-helices as secondary structures. In addition, conservation analysis showed that mutations of 100% conserved residues at positions 144 and 148 were probably harmful to the protein. The structural visualization of the four most detrimental missense SNPs was conducted using the HOPE server, as illustrated in Fig. 3.

The secondary structure and percentages of the alpha helices, coils, and extended strands, in the OX1R native structure, were evaluated using the SOPMA tool as mentioned in Table S3. SOPMA revealed that 199 residues were associated with alpha helix (46.82%), 53 with extended strand (12.47%), 14 with beta-turn (3.29%), and 159 with the random coil (37.41%). Moreover, an analysis of the most detrimental SNPs’ effects on the alterations in the secondary structure of the OX1R protein was conducted as mentioned in Table S3. It was observed that in all mutant proteins, a slight reduction in the number of residues participating in the alpha helix conformation was accompanied by a slight increase in the extended strand conformation compared to the native structure.

### Molecular dynamics simulations

MDs measurements for the four most detrimental missense SNPs (R144C, I148N, S172W, and A297D) were conducted for 200 ns simulations to characterize the structural perturbation of OX1R protein caused by these missense SNPs in normal physiological conditions.

### Stability analysis

**Figure 4:**
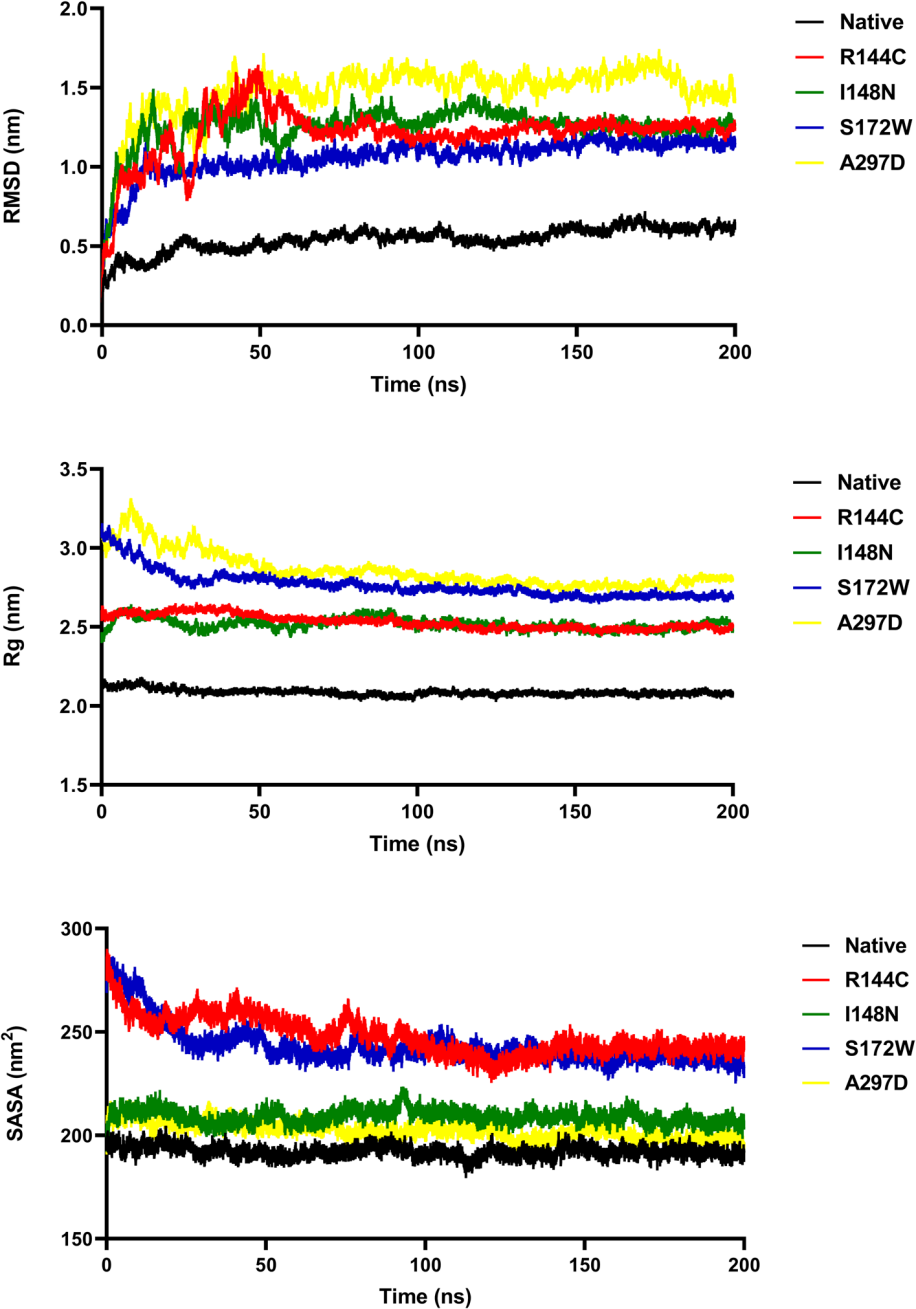
Analysis of RMSD, Rg, and SASA of native and four mutants of OX1R over 200 ns simulation. (**A**) RMSD values of Cα atoms of native and mutant structures. (**B**) Calculation of Rg, which is represented as a time-dependent change during the simulation. (**C**) Calculation of SASA represented as a time-depend. The color scheme is as follows: native (black color), R144C mutant (red color), I148N mutant (green color), S172W (blue color), and A297D mutant (yellow color).

The RMSD value was estimated to comprehend the overall stability of protein during the simulation^46^. RMSD of the backbone’s atoms of the native structure and the mutant proteins were plotted against the time to evaluate the alteration effects (Fig. 4A). Compared to the native structure, a significant increase in the average RMSD values for the R144C, I148N, S172W, and A297D mutant proteins was observed (Fig. 4A). The average RMSD for the native protein was found to be 0.542 nm, while for mutants R144C, I148N, S172W, and A297D, they were found to be 1.211 nm, 1.251 nm, 1.061 nm, and 1.473 nm, respectively. A higher RMSD value indicates a reduction in protein stability. Since stability is an important aspect of protein activity and function, decreased stability may have a damaging impact on the overall function of the protein^46^. Data showed that all mutants showed higher RMSD value and fluctuation during molecular dynamics simulation which means the unstable structure of mutants compared to the native protein.

### Compactness analysis

The Rg value indicates the overall dimension of the protein, thus acting as a crucial marker for assessing protein structure compactness^47^. To analyze the impact of the four most detrimental missense SNPs on the structural integrity of OX1R, the Rg value was determined for native and mutant proteins.

As shown in Fig. 4B the mutants R144C, I148N, S172W, and A297D showed significant divergence in the structural compactness and rigidity of the native protein. The native protein had an average Rg value of 2.085 nm, whereas the mutants R144C, I148N, S172W, and A297D had average Rg values of 2.530 nm, 2.518 nm, 2.763 nm, and 2.857 nm, respectively. The results indicated that all mutants had fewer compact and more flexible structures, as their Rg values were higher compared to the native structure.

### Solvent accessible surface area analysis

To determine the effect of the four most detrimental missense SNPs on the compactness of the hydrophobic core of the OX1R protein, SASA analysis was conducted. As displayed in Fig. 4C an alteration in SASA between the native and mutant proteins was observed. The average SASA values for the mutants R144C, I148N, S172W, and A297D were 247.796 nm^2^ 209.098, 243.33 nm^2^, and 202.499 nm^2^, respectively, whereas, for the native structure, it was 192.189 nm^2^. A greater SASA value signifies an expansion of a protein, suggesting that all mutant proteins may achieve more expanded structures compared to the native protein.

### Flexibility analysis

**Figure 5:**
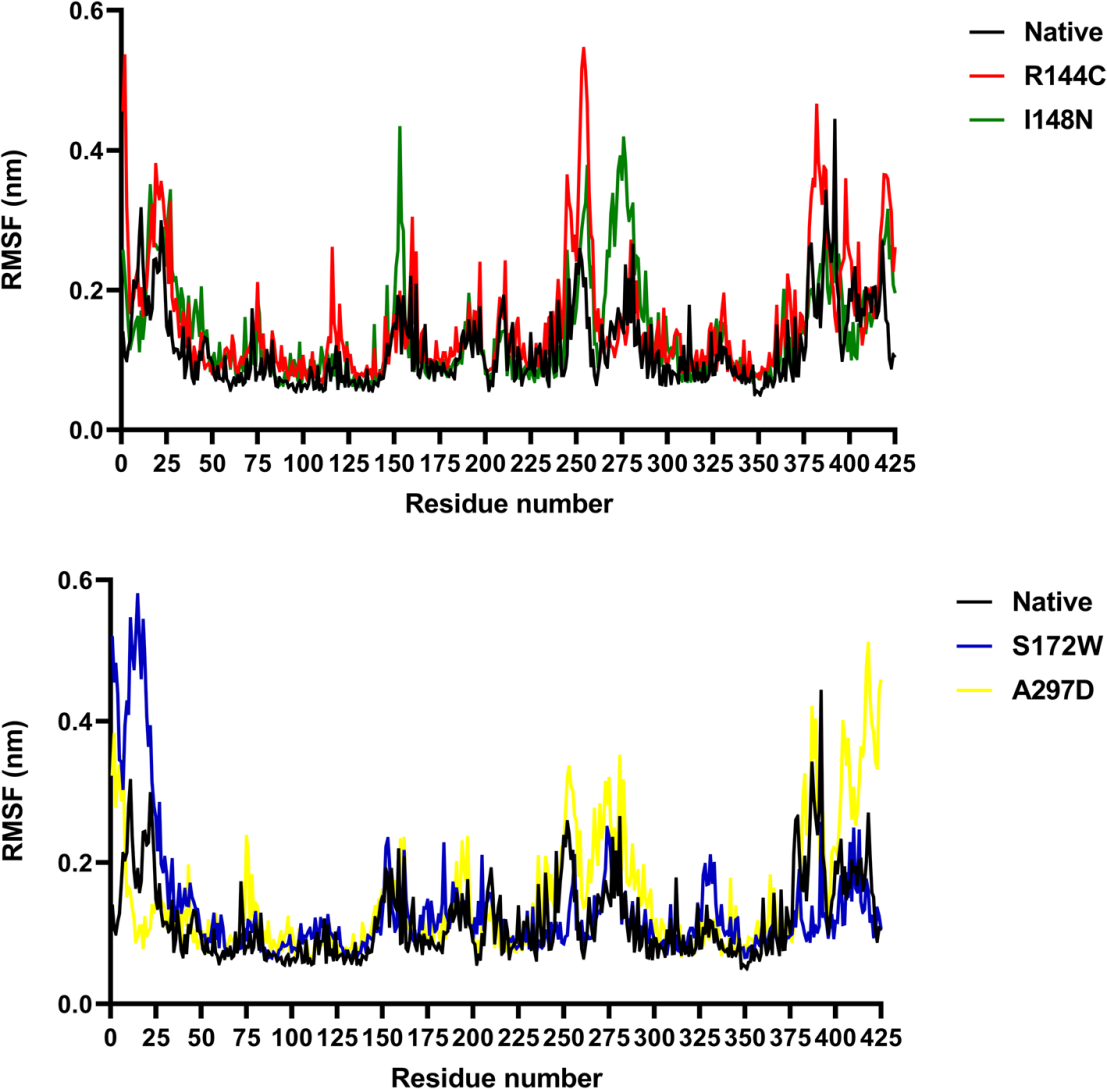
RMSF of Cα atoms of the native structure and mutant proteins over 200 ns simulation. (**A**) RMSF plot of native structure (black color) vs. R144C (red color) and I148N (green color) mutant proteins. (**B**) RMSF plot of native structure (black color) vs. S172W (blue color) and A297D (yellow color) mutant proteins.

To predict the impact of the four most detrimental missense SNPs on the dynamic behavior of residues within the OX1R protein structure, RMSF analysis was performed. The RMSF value is essential for understanding the overall flexibility of proteins during simulation. The backbone RMSF for each residue number was estimated for the native and mutant proteins (Fig. 5). The native protein had an average RMSF value of 0.117 nm. For mutants R144C, I148N, S172W, and A297D, the average RMSF values were 0.154, 0.139, 0.134, and 0.148 nm, respectively. Hence, slightly higher flexibility was observed for all mutant proteins compared to that of the native protein. Mutants R144C and I148N, compared to the native structure, showed higher fluctuations between residues 245 to 260 and 250 to 280, respectively (Fig. 5A). The S172W mutant showed a prominent peak of 0.58 nm at the very first residues (Fig. 5B). In mutant A297D, residues 250–280 and 400–425 were observed to have higher flexibility than the native structure (Fig. 5B). According to RMSF analysis, although there were no significant changes in the overall flexibility of all mutant proteins, residual changes were observed.

### Secondary structure analysis

**Figure 6:**
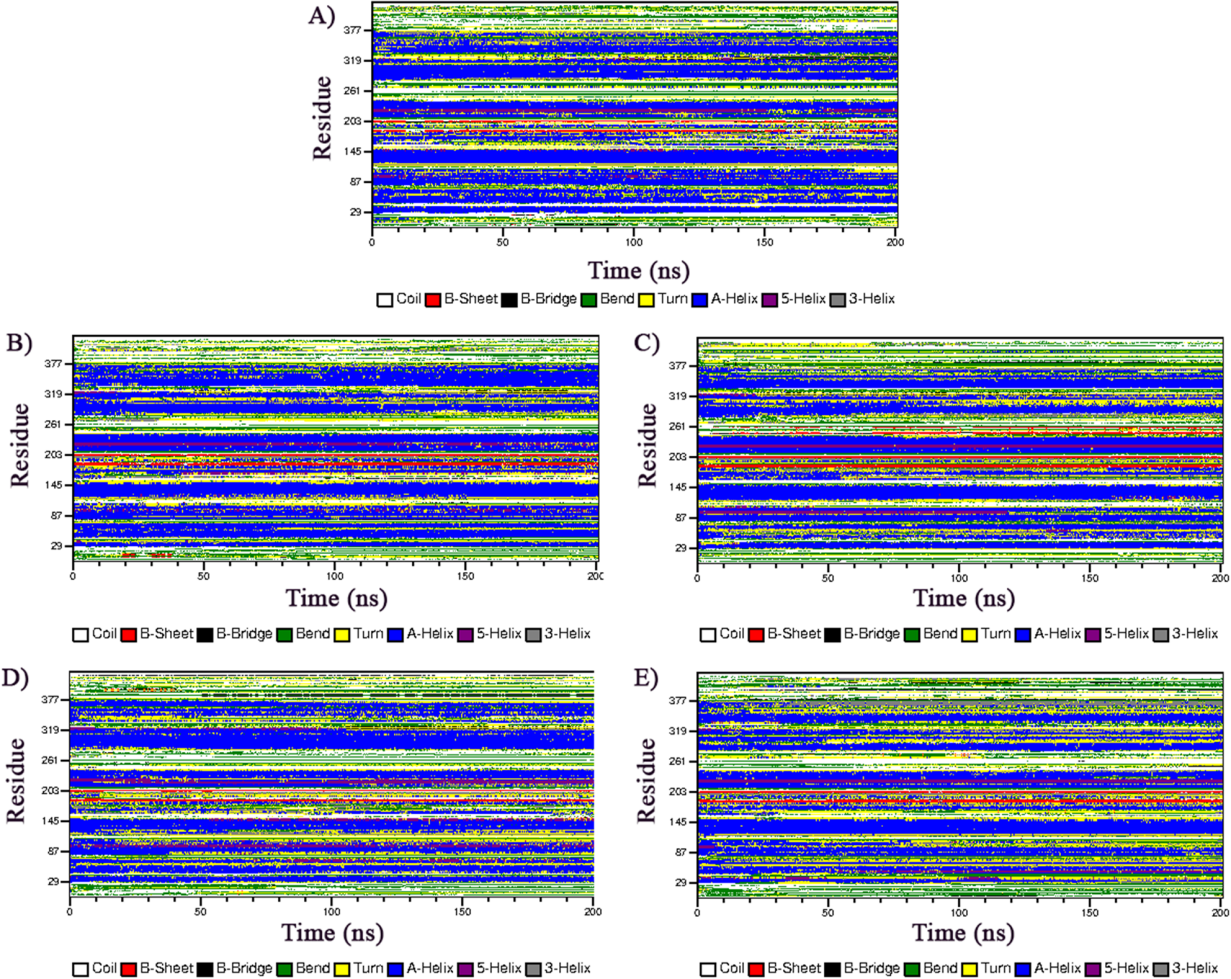
Effect of the four most missense SNPs in the stability of the secondary structure elements of the OX1R during the 200 ns simulation. (**A**) native structure, (**B**) mutant R144C, (**C**) mutant I148N, (**D**) mutant S172W, and (**E**) mutant A297D.

To identify the alteration in OX1R protein secondary structural arrangement caused by the four most detrimental missense SNPs, the secondary structure compositions of native and mutant proteins were analyzed, as shown in Fig. 6. The mutant R144C (Fig. 6B) showed a slight increase in the β-sheet content at residue 185 to 203, the other elements were marginally similar to the native protein. The mutant I148N showed a tiny increase in the β-sheet trend at residues 185 to 203 and 185 to 261, while a transition from α-helix to bend and turn to residues 300 to 330 compared to the native protein (Fig. 6C). The S172W mutant showed α-helices turning into turns, bends, and 5-helices between residues 85–120 and an increase in the β-sheet trend at residues 185 to 203 compared to the native protein (Fig. 6D). In mutant A297D, many α-helices turned into bends, turns, 5-helices, and 5-helices between residues, 30–120 and 280–360. Furthermore, an increase in the β-sheet content at residue 185 to 203 compared to the native protein also was observed (Fig. 6E).

The evaluation of the percentage of each secondary structure in native and mutant proteins showed a moderate reduction in the structural profile of mutants R144C, I148N, S172W, and A297D compared to the native structure (Table S4). Significant alterations in the secondary structure composition of all mutants can be found as a result of inhibiting the α-helix conformation while increasing the B-sheet conformation compared to the native protein, which is in line with the SOPMA outcomes shown in Table S3, where the number of α-helixes residues were altered in all mutants.

### Principal component analysis

**Figure 7:**
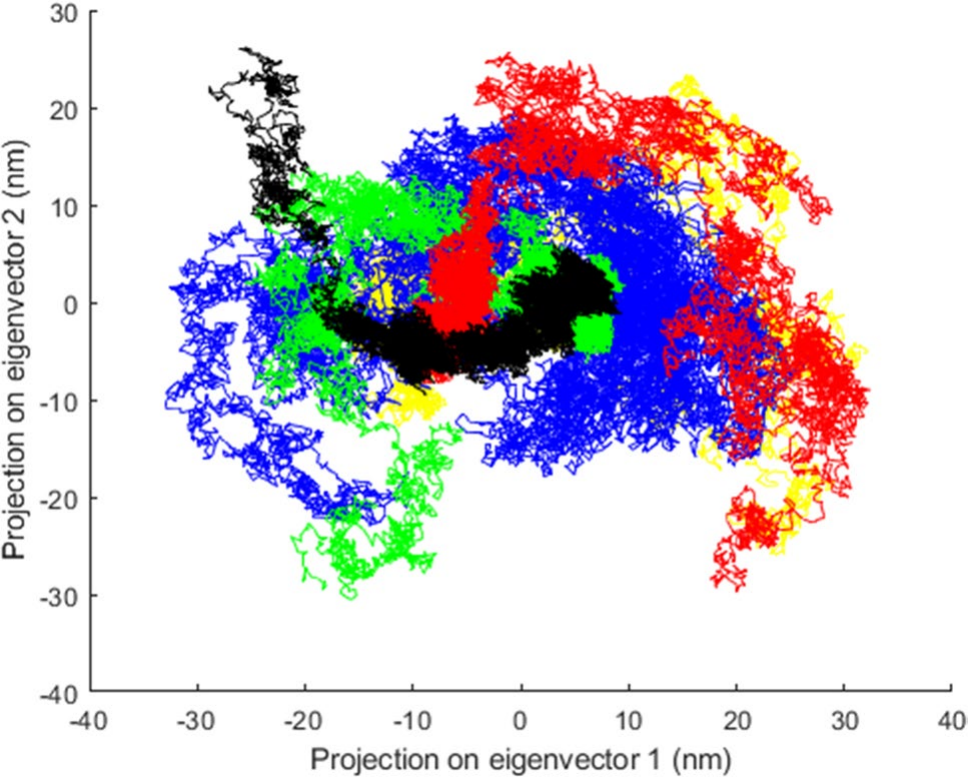
The principal component analysis (PCA) of the native structure and mutant proteins over 200 ns simulation. The color scheme is as follows: native (black color), R144C mutant (red color), I148N mutant (green color), S172W (blue color), and A297D mutant (yellow color).

PCA was conducted to determine the effect of the four most detrimental missense SNPs on structural motion and locally restricted fluctuations of native and mutant proteins. Principal components (PCs) with larger eigenvalues are eigenvectors that play an important role in the overall concerted motion of the protein^48^. The first two PCs (PC1 and PC2) were chosen to determine the collective motion in the phase space during the simulation. As depicted in Fig. 7 mutants R144C, I148N, S172W, and A297D occupied more space than the native protein. The PCA findings suggested that the concerted motions were perturbed after mutations, resulting in the loss of structural stability in mutant proteins, in agreement with RMSD, Rg, SASA, RMSF, and secondary structural analyses.

### Free energy landscape analysis

**Figure 8:**
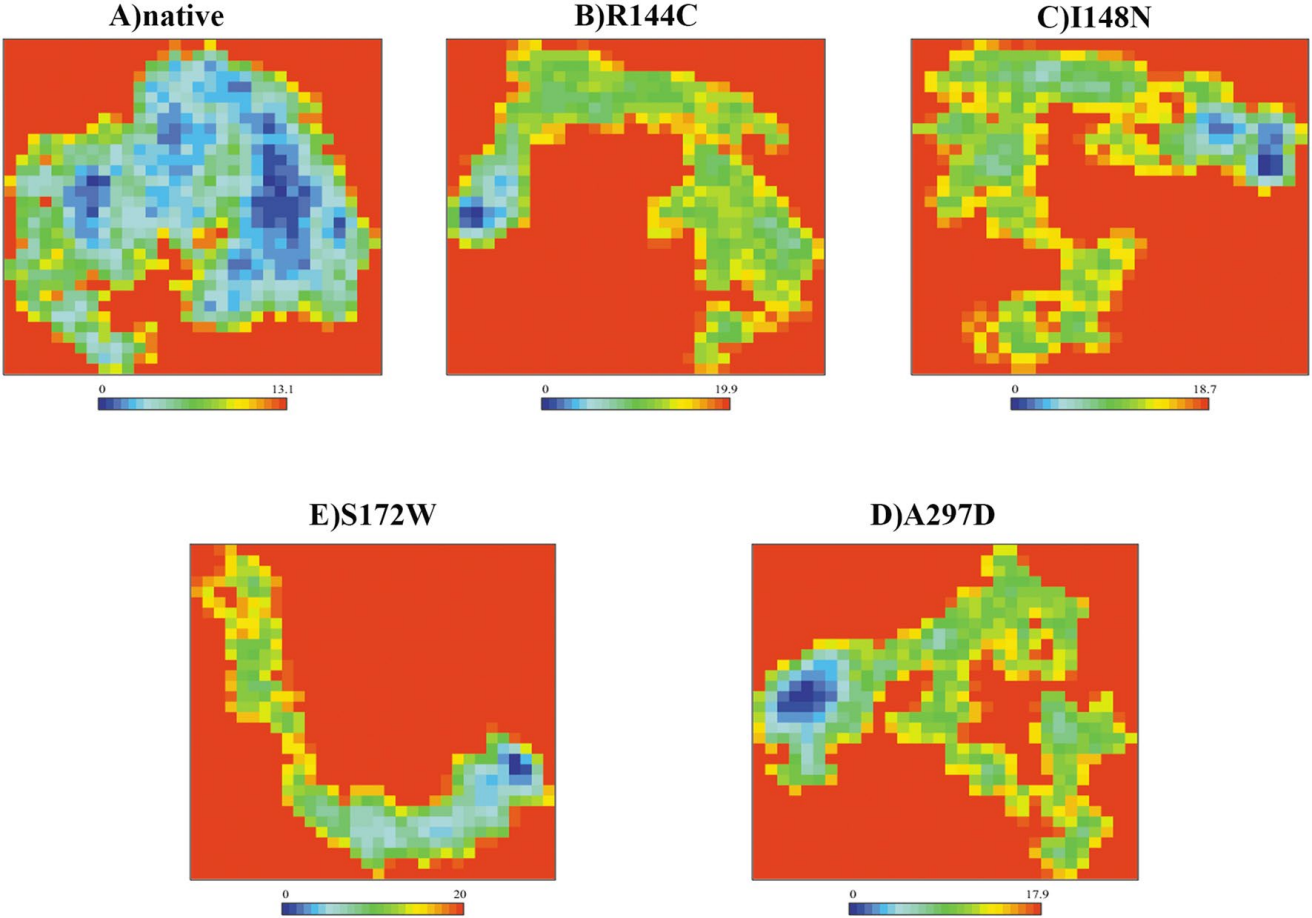
Free energy landscape (FEL) of the native structure and mutant proteins over 200 ns simulation. (**A**) native structure, (**B**) mutant R144C, (**C**) mutant I148N, (**D**) mutant S172W, and (**E**) mutant A297D.

Using the first two eigenvectors of the FELs, all possible conformation of atoms, present in native and mutant proteins, were explored. FEL color spectrum labels range from blue to red, where blue spots indicate folds and the highest stable state of the protein, while red spots represent unfolded states and a lower stable state. Figure 8 illustrates that all mutants R144C, I148N, S172W, and A297D exhibited fewer blue spots with structure expansion and a greater range of Gibbs free energy than the native protein. As the folding pattern of a protein directly affects its stability, more unfolded states suggest a decrease in the stability of the protein structure resulting from the four most detrimental missense mutations. The results obtained by FEL analysis were consistent with those obtained by RMSD and Rg analysis.

### Determining the effect of mutations on OX1R interaction

**Figure 9:**
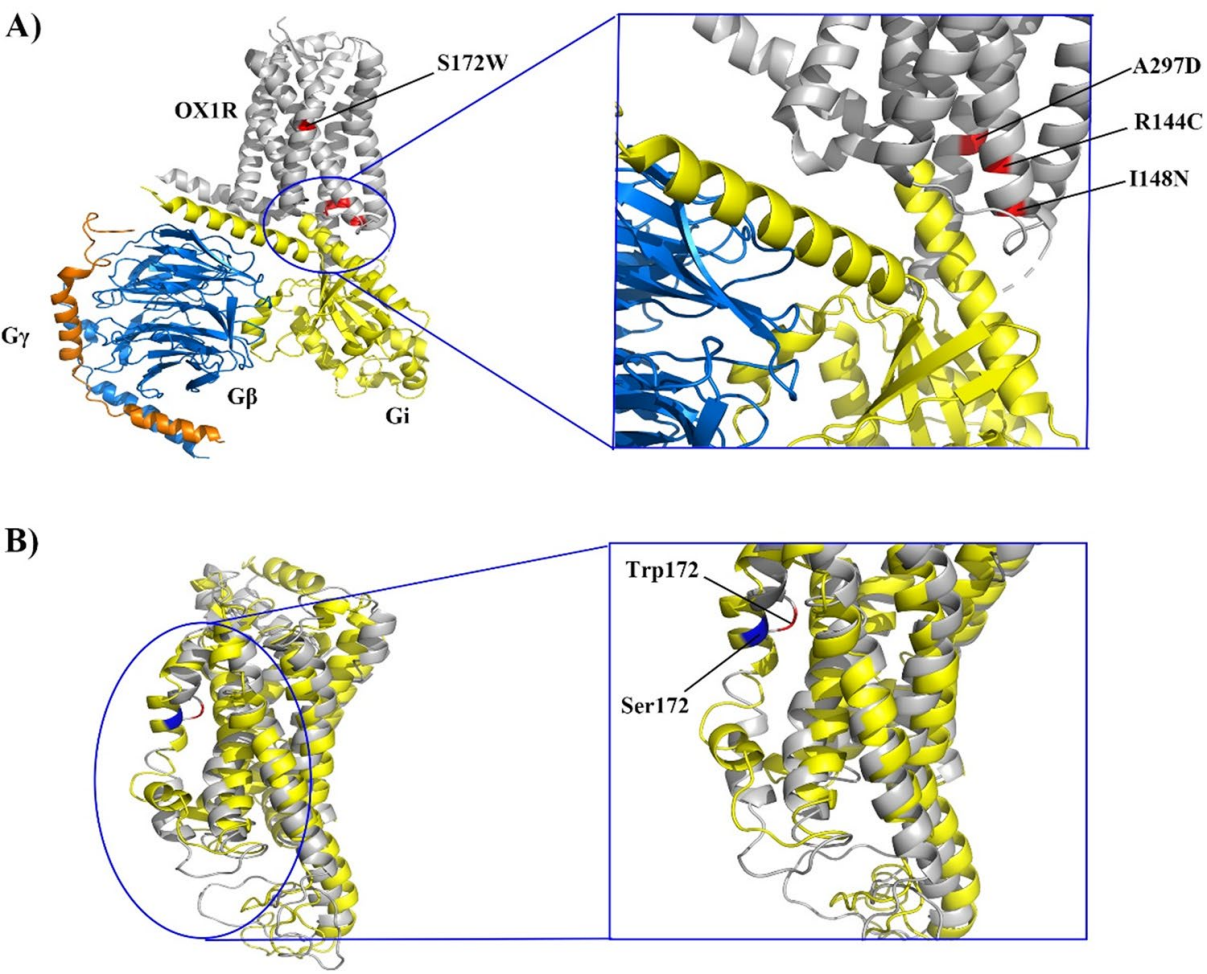
Structure analysis of OX1R mutant forms. (**A**) The mode of OX1R interaction with Gi protein. Data shows that mutant forms of I148N, A297D, and R144C except S172W are located in regions that directly interact with Gi protein. (**B**) The structural alignment of native(yellow) and mutant(gray) forms of OX1R after simulation. The result shows that changing Ser172 (red) to Trp caused changes in the alpha helix structure which is located transmembrane domain of the OX1R.

Previous studies have demonstrated that OX1R belongs to the GPCR protein family, which interacts with G protein complexes composed of Gi, Go, Gs, or G11 to transmit signals intracellularly^49^. Therefore, any changes in amino acids in these regions may affect the interaction between OX1R and Gi protein. The structure analysis of OX1R mutants demonstrated that mutants R144C, I148N, and A297D except for S172W located in the region could affect OX1R signaling by changing the structure and the binding mode with Gi protein (Fig. 9A). In addition, the structure analysis of S172W after simulation demonstrated that this mutation altered the structure of the transmembrane alpha-helix and affected the position of OX1R in the membrane (Fig. 9B).

Protein–protein interaction analysis demonstrated that all four detrimental missense SNPs affected OX1R interaction with the Gi protein (Table S5). The data obtained from free binding energy calculations using the HAWDOCK web server, ∆G assessments with Prodigy, and the predictions of affinity changes (∆∆G) from the mCSM-PP2 tool collectively illustrate that mutants A297D and S172W had significantly lower free binding energy than the native, while mutants I148N and R1444C had slightly higher free binding energy (Table 3). These results suggest that the complexes formed by mutants A297D and S172W exhibit greater stability with the Gi protein than those formed by I148N and R144C.

**Table 3:** Protein–protein interaction analysis of OX1R with Gi protein.

Model	HAWDOCK(MM/GBSA)					Prodigy		mCSM-PPI2	
	Free binding energy	VDW	ELE	GB	SA	ΔG (Kcal/mol)	Affinity	ΔΔG (kcal/mol)	Effect of affinity
Native	− 53.73	− 115.6	− 829.9	907.5	− 15.6	− 10.7	1.3e−08	–	–
I148N	− 50.43	− 97.68	− 967.24	1028.61	− 14.12	− 10.6	1.8e−08	− 0.113	Decrease
A297D	− 112.01	− 223.4	− 818.7	960.8	− 30.5	− 18.1	5.2e−14	0.318	Increasing
R144C	− 47.4	− 94.5	− 833.6	893.5	− 12.7	− 9.6	8.7e−08	− 0.462	Decrease
S172W	− 88.19	− 180.54	− 1070.17	1187.11	− 24.6	− 16.8	4.8e−13	0.347	Increasing

*VDW* Van der Waals potentials, *ELE* Electrostatic potentials, *GB* Polar solvation free energies, *SA* Nonpolar contribution to the solvation-free energy.

## Discussion

Accumulation of data from numerous studies has demonstrated the contribution of various missense SNPs to the progression of diverse diseases^36,37,40^. However, there is a paucity of knowledge regarding the alterations in OX1R structural and conformational dynamics caused by detrimental missense SNPs. In this study, we carried out a comprehensive in silico evaluation to forecast pathogenic SNPs and their potential impacts on the structure and function of OX1R protein by employing a combination of different computational approaches. To enhance prediction accuracy, we integrated tools from diverse categories, including homology-based, sequenced-based, consensus-based, and structure-based approaches. This strategy aims to increase confidence in the identification of potentially detrimental missense SNPs by mitigating biases in the results^50^.

By filtering 4,295 SNPs located in the OX1R gene, a total of 329 missense SNPs were identified. Further evaluation in two steps, using 15 bioinformatics tools, revealed that 17 missense SNPs were pathogenic to OX1R. All isolated 17 missense SNPs were found in the GTP-binding domain of the protein. This domain is responsible for catalytic activity. Therefore, mutations in this region are expected to affect protein activity and function^4,31^.

In addition, stability analysis indicated that five SNPs (R144C, I148N, S172W, A297D, and L312Q) among the 17 pathogenic SNPs could lead to a decrease in OX1R stability, based on the results of all seven prediction tools. Protein stability is a critical characteristic that affects its structure, function, evolution, and biological activity, as well as its normal pathways^50^. Any modification of protein stability may contribute to aberrant accumulation, misfolding, or protein degradation^51^.

Conservancy evaluation revealed that four of the five SNPs (R144C, I148N, S172W, and A297D) were found at crucial sites in the protein, as their conservation scores were high. The conservation level of a residue exhibits a strong correlation with its functional and structural significance. Generally, SNPs occurring in positions that are evolutionarily conserved are not well-tolerated, thereby resulting in the progression of diseases^52,53^.

Hence, the four mutants, namely, R144C, I148N, S172W, and A297D, were identified as the most detrimental missense SNPs of the OX1R protein and selected for further computational analysis to determine their impact on the structure and function of the OX1R protein.

The structural impact of the four selected SNPs (R144C, I148N, S172W, and A297D) was assessed using the HOPE server. The R144C mutant leads to the substitution of arginine (positively charged residue) by cysteine (neutrally charged), where arginine is larger than cysteine. Charge and mass differences in proteins influence protein–protein spatiotemporal dynamics. The I148N, S172W, and A297D mutants had a larger residue than the wild-type moiety, which likely did not fit into the protein core. Consequently, these SNPs cause structural changes that are sometimes hazardous. Moreover, the wild-type residues of I148N, S172W, and A297D were more hydrophobic than the mutant residues, which could have caused repulsive hydrophobic interactions in the core of the protein, as predicted by the HOPE server. Generally, structural mutations cause changes in amino acid charge and size, hydrophobic propensity, salt bridges, and hydrogen bonds, which could cause loss of thermodynamic stability as well as aberrant folding and aggregation of proteins.

Secondary structure analysis conducted using the SOPMA server revealed that the most detrimental missense SNPs (R144C, I148N, S172W, and A297D) exhibited a modest decrease in the number of residues involved in the alpha-helix conformation, accompanied by a slightly higher proportion of extended strand conformation compared to the native structure. This observation suggests that these mutations may have a detrimental effect on OX1R protein stability.

Proteins possess a dynamic nature. We conducted 200 ns MDs to investigate the consequence of mutants R144C, I148N, S172W, and A297D on the structural dynamics and stability of the OX1R protein. The plateau of RMSD values revealed a significant decrease in protein stability for the mutants R144C, I148N, S172W, and A297D compared to the native structure throughout the MDs. Furthermore, a loss of stability and higher residue fluctuation was observed for all four mutant proteins compared to the native structure in the Rg and RMSF analysis. In support of these findings, SASA analysis showed that the high flexibility induced by all mutants may lead to expansion of the solvent-exposed area, leading to protein misfolding and being regarded as accountable for the loss of function. The secondary structure analysis also pointed out that the mutants R144C, I148N, S172W, and A297D compared to the native structure, introduced a slightly more β-strand conformation, whereas they disrupted the α-helix conformation, especially for the A297D mutant. These deviations might have an impact on protein folding thereby decreasing the stability of the protein. Results derived from the PCA confirmed that all four mutant proteins enhanced the overall flexibility and motion of the OX1R protein. Moreover, from FEL energy analysis, all mutants R144C, I148N, S172W, and A297D showed a significant difference in the stability of than native protein. A change in the protein stability could alter the protein function^54^. Therefore, we indicate that the four most detrimental mutant proteins, R144C, I148N, S172W, and A297D might exhibit a significant effect on OX1R structure and function. This supposition is well-aligned with the outcomes derived from the investigations conducted by Saxena et al.^55^, Agrahari et al.^56^, and Shinwari et al.^57^.

Signaling through OX1R is well established for its role in neuronal depolarization, which enhances excitability and firing rates through the activation of a non-selective cation current. Moreover, the orexin/OX1R system has been linked to inducing mitochondrial apoptosis, leading to a substantial reduction in cell growth across various cancer lines^58,59^. An analysis of the structure positioning of the most detrimental mutant proteins on OX1R, in comparison with the µ-opioid protein complex as a GPCR receptor, revealed that the mutants R144C, I148N, and A297D are situated in regions directly interacting with the Gi protein^60^. Protein–protein interaction studies further demonstrated that mutants A297D and S172W exhibited increased binding affinity with the Gi protein compared to the native form, while mutants I148N and R144C showed decreased binding affinity. The observed alterations in binding affinity with the Gi protein, as indicated by mutants R144C, I148N, S172W and A297D, suggest changes in Gi protein activation and subsequent downstream signaling pathways of OX1R. These structural changes have the potential to influence the regulation of homeostatic processes and antitumor activity associated with OX1R^61,62^. Although S172W is not in close proximity to the Gi protein-binding site, secondary structure predictions suggest that it could induce conformational changes in OX1R protein, affecting its interaction with the Gi protein. This finding aligns with the work of Hong et al., indicating that interaction with extracellular region of OX2R can induce local and global conformational changes, impacting receptor activation^49^.

Our study identified pathogenic missense SNPs in the OX1R structure and explore their functional consequences, which can be further verified by experimental analysis to determine their role more precisely. However, insights into the structural positioning of detrimental mutants and their effects on Gi protein interactions offer opportunities for the development of targeted drugs, precision medicine strategies, and novel approaches to neurological disorders and cancer therapeutics.

## Conclusion

In the current study, we employed a combination of in silico tools, MDs, and protein–protein interaction analyses to identify the most deleterious missense mutations affecting the structure and function of the OX1R protein. We detected 17 pathogenic mutations among 329 missense SNPs within the *OX1R* gene, primarily located in the GTP-binding domain. Four highly conserved missense SNPs, namely R144C, I148N, S172W, and A297D among the pathogenic SNPs were identified as significantly altering the dynamic stability and structure conformation of OX1R. Our computational pipeline offers a promising approach for prioritizing potential therapeutic targets, particularly for neurological disorders and cancer therapeutics associated with OX1R mutations.

## Methods

### Data retrieval

The list of all SNPs related to the *OX1R* gene was collected from the National Center for Biotechnology Information (NCBI) dbSNP database and mapped on genome assembly GRCh37.p13 (hg19) through the utilization of Variation Viewer^63^. “OX1R” or “HCRTR1” was utilized as our search keyword and filtered for SNPs (https://www.ncbi.nlm.nih.gov/variation/view/?q=HCRTR1). The human OX1R protein's FASTA sequence was obtained from the UniProt database (UniProt ID: O43613)^64^.

### Determining 3D protein structure and its validation

As the complete structure of the OX1R protein is not found in the PDB bank, homology modeling was performed by the Robetta web server (https://robetta.bakerlab.org/)^65^. To validate the tertiary structure of the OX1R model, the SAVES web server which compromised ERRAT, Verify3D, and Procheck tools was applied (https://saves.mbi.ucla.edu/)^66^. The ERRAT web tool computes the quality of non-bonded interaction. The ERRAT value of around 91% to 95% means good quality protein structure^66^. The Verify3D web server investigates the compatibility of the secondary structure of a protein based on one-dimension (1D) with the three-dimensional (3D) structure of the protein structure. The Verify3D value of more than 80% illustrates compatibility between the two structures^67^. The Procheck web tool analyzes the stereochemical quality of each residue geometry in protein structure according to the Ramachandran plot^68^. Based on this web server, the higher percent of residues located in the core and generally allowed region indicate a higher quality of protein structure. The Molprobity web server calculates the resolution of protein structure by combing and evaluating the clash score, rotamer, and Ramachandran into a single score and normalizing the scale as an X-ray resolution^69^. The higher Molprobity score illustrates the better quality of protein structure. The ProSA web server evaluates the quality of protein structures by comparing their quality with protein structures whose structure was identified by X-ray and NMR^70^. Structural representation of human OX1R protein was done by using PyMOL software.

### Determining the pathogenic nsSNPs

We used a multistep strategy employing various computational algorithms to identify deleterious nsSNPs in the human *OX1R* gene. In the first step, we predicted the functional effects of the nsSNPs on the protein using 10 different in silico prediction tools. These tools included FATHMM-MLK^71^, SIFT^72^, Mutation assessor^73^, SNAP-2^74^, Polyphen-2^75^, Panther-PSEP^76^, PON-P2^77^, CADD^78^, Align-GVGD^79^, and VEST-4^80^. In the next step, we utilized five computational tools, namely Pmut^81^, Suspect^82^, PhD-SNP^83^, SNPs&GO^84^, and InMeRF^85^, to assess the pathogenicity and disease association of the identified nsSNPs.

### Determining the domain of OX1R protein

To ascertain the domains and positions of disease-causing nsSNPs in the OX1R protein, four databases, namely InterPro^86^, PROSITE^87^, Pfam^88^, and CDD^89^, were used. For all four tools, the FASTA sequence of the protein was provided as input.

### Determining SNP’s impact on the OX1R protein stability

To understand whether the OX1R protein will be in a stable or denatured form due to amino acid substitutions, we applied eight different in silico algorithms: three sequenced-based tools, I-Mutant 2.0^90^, INPS-MD^91^, Mu-Pro^92^, and five structure-based servers, m-CSM^93^, SDM^94^, DUET^95^, and Dyna-Mut^96^.

### Determining the phylogenetically conserved residues in the OX1R protein

The evolutionary pattern of conservation at each amino acid position in OX1R was determined using the ConSurf server. For each residue of the target protein, the level of conservation was calculated on a scale of 1 to 4 as a variable, 5–6 as an average, 7–8 as conserved, and 9 as extremely conserved^97,98^. Functional and structural residues have also been identified. The 3D structure of the OX1R protein was used for ConSurf evaluation.

### Determining the phenotypic and structural consequences of selected SNPs

To further investigate the structural and functional effects of amino acid modifications on the OX1R protein, we utilized the HOPE server^99^.

Alterations in the secondary structure of the OX1R protein due to pathogenic SNPs, based on the amino acid sequences of the native and mutant proteins, were provided by the SOPMA server^100^.

### Molecular dynamics simulations

To analyze the effect of each mutation on protein structure changes, molecular dynamics simulations (MDs) were performed for native and mutant forms by Gromacs 2020.7^101^. The OPLSA force field was applied during the simulation. A cubic simulation box was filled with SPCE water type. The simulation system was neutralized with Na^+^ and Cl^-^. The system was energy minimized by the steep descent minimization algorithm for a maximum of 50,000 steps. For equilibration of the simulation system, a Berendsen temperature (tcouple) of 300 K and a Parrinello-Rahman pressure (pcouple) of 1 bar were selected. The electrostatic energy was computed by the Partial Mesh Ewald (PME) algorithm and the LINCS algorithm was applied to constrain all bonds. MDs were performed for 200 ns and the trajectory was given at intervals of 1 ps. The trajectory was used to analyze the structure changes by Root Mean Square Deviation (RMSD), Gyrate (Rg), Root Mean Square Fluctuation (RMSF), Hydrogen bond (H-bond), Solvent Access Surface Area (SASA), Principal component analysis (PCA), and Free energy landscapes (FELs) for both native and mutant forms. To compute the secondary structure changes during MDs, DSSP software was used.

### Determining the effect of mutations on OX1R interaction

To analyze the effect of mutations on the activation of the OX1R pathway, protein–protein interactions were performed using the PPI3D web server^102^. To do this, the conformation mode of PDB ID:7u2k of C6-guano bound Mu Opioid Receptor-Gi Protein Complex was applied. The results of the PPI3D tool were assessed using the Ligplot package to illustrate the amino acids that play a role in the interactions^103^. Furthermore, the HAWDOCK tool was used to determine the types and total free-binding energies of the protein–protein complexes. The binding affinity of the protein–protein complex was calculated using the Prodigy server. The effect of each mutation on protein–protein was studied by the mCSM-PPI2 tool^104^.

### Supplementary Information

Supplementary Figure S1.

Supplementary Figure S2.

Supplementary Figure S3.

Supplementary Table S1.

Supplementary Table S2.

Supplementary Table S3.

Supplementary Table S4.

Supplementary Table S5.

## Data Availability

The datasets used and/or analyzed during the current study are available from the corresponding author upon reasonable request.
