# Supplementary material for: Unraveling the function and structure impact of deleterious missense SNPs in the human OX1R receptor by computational analysis
Source: Sci Rep. 2024 Jan 8;14:833. doi: 10.1038/s41598-023-49809-4 (PMC10774445; doi:10.1038/s41598-023-49809-4)
Supplement: Supplementary file 8 — Supplementary Table S5. [file 41598_2023_49809_MOESM8_ESM.docx]

**Unraveling the Function and Structure Impact of Deleterious Missense SNPs in the Human OX1R Receptor by Computational Analysis**

Mahvash Farajzadeh-Dehkordi ^1&2^, Ladan Mafakher ^3^, Abbas Harifi ^4^, Hashem Haghdoost-Yazdi^5^, Hossein Piri ^5^ & Babak Rahmani ^1&2*^

1. Student Research Committee, Qazvin University of Medical Sciences, Qazvin, Iran.
2. Department of Molecular Medicine, Qazvin University of Medical Sciences, Qazvin, Iran.
3. Thalassemia & Hemoglobinopathy Research Center, Health Research Institute, Ahvaz Jundishapur University of Medical Sciences, Ahvaz, Iran
4. Department of Electrical and Computer Engineering, University of Hormozgan, Bandar Abbas, Hormozgan, Iran.
5. Cellular and Molecular Research Center, Research Institute for prevention of Non- Communicable Disease, Qazvin University of Medical Sciences, Qazvin, Iran

Correspondence: [b.rahmanigene@gmail.com](mailto:b.rahmanigene@gmail.com)

**Table S5.** Protein-protein interaction analysis of each mutant and native protein by Ligplot.

| model | orexin | Gi protein |
| --- | --- | --- |
| Native | Arg245, Ile148, Lys241, Ala147, Thr81, Pro151, Arg159, Ser362, Arg281, Gln277, Pro276, Ala280, Pro278, Arg279, Arg144, Met299, Gln246 | Leu348, Ile344, Asn347, Cys351, Ile343, Arg24, Gly352, Arg313, Lys312, Lys314, Phe35, Asp315, Thr316, Leu353, Tyr353, Tyr320, Glu318 |
| I148N | Arg245, Leu152, Ala147, Asn148, Lys241, Ser362, Lys364, Arg159, Arg281, Ala180, Arg279, Leu153 | Leu348, Ile343, Asn347, Ile344, Gly352, Cys351, Asp350, Lys349, Arg24, Asp315, Thr316, Glu318, Lys317, Lys314, Ala31, Arg32 |
| A297D | Lys259, Ser262, Pro261, Arg260, Thr250, Thr251, Glu286, Thr81, Ile148, Ala147, Arg245, Gln246, Ile247, Pro151, Arg159, Met299, Arg256, Asn257, Val255, Trp285, Ala252, Leu254 | Ala291, Gly292, Glu297, Glu308, Glu298, Tyr302, Gln306, Cys305, Tyr320, Ile319, Thr321, His322, Asp315, Cys351, Ile344, Asn347, Asp341, Ile343, Arg24, Leu253, Lys279, Ser293, Glu275, Tyr296, Thr295, Asn294, Ile278, Phe274, Ala299, Ser281, Pro282, Thr284, Leu283, Ile285, Ala300, Phe323, Gln304, Ile303, Ala301, Tyr290 |
| R144C | Phe154, Leu153, Ala147, Gln246, Met299, Thr81, Arg254, Ile148, Arg159, Leu272, Gln269, Leu268, Asp267, Gln270 | Arg32, Asn347, Asp341, Tyr320, Leu353, Cys351, Ile344, Arg24, Leu348, Phe354, Thr316, Asp315, Lys314, Lys317 |
| S172W | Pro278, Ala285, Val287, Lys288, Gln277, Glu286, Lys295, Arg245, Pro151, Ala147, Ile148, Thr81, Arg159, Arg281, Ala282, Ala280, Arg279, Phe283, Leu284 | Phe259, Asp261, Thr316, Asp315, Lys349, Phe354, Lys345, Leu353, Tyr320, Asp341, Ile343, Asn347, Ile344, Leu348, Asp350, Asn311, Lys317, Arg313, Thr262, Asn256, Trp258, Lys314, Thr260, Lys257 |
